# Shared Mechanism, Distinct Outcomes: Transcriptomic Analysis Reveals Differential Modulation of Metabolic and Detoxification Pathways by Neonicotinoid Insecticides

**DOI:** 10.3390/ijms27114785

**Published:** 2026-05-26

**Authors:** Gabriel Colissi-Martins, Fernanda Mocellin Conte, Marcelo Dutra Arbo

**Affiliations:** 1 Instituto de Ciências Básicas da Saúde, Universidade Federal do Rio Grande do Sul, Porto Alegre 90035-003, RS, Brazil; gabrielcolmar7@gmail.com; 2 Laboratório de Toxicologia, Departamento de Análises, Faculdade de Farmácia, Universidade Federal do Rio Grande do Sul, Porto Alegre 90620-170, RS, Brazil; fconte@universo.univates.br; 3 Programa de Pós-Graduação em Ciências Farmacêuticas, Faculdade de Farmácia, Universidade Federal do Rio Grande do Sul, Porto Alegre 90610-000, RS, Brazil

**Keywords:** neonicotinoids, pesticides, transcriptomics, clothianidin, thiacloprid, toxicology

## Abstract

Neonicotinoids are among the most widely used classes of insecticides worldwide. However, growing evidence links their exposure to metabolic disturbances, including DNA damage, endocrine disruption, and hepatic dysfunction. In this study, transcriptomic analyses were applied to investigate the gene expression changes induced by two neonicotinoids, clothianidin and thiacloprid. Our results revealed distinct treatment-driven transcriptional signatures, characterized by the upregulation of gene sets enriched in pathways associated with mitochondrial regulation, neuronal signaling, and neurodegeneration-related molecular processes, alongside the downregulation of genes involved in core metabolic processes. In addition, neonicotinoid exposure modulated gene sets associated with xenobiotic detoxification, immune response, cell proliferation, and cell adhesion. Notably, clothianidin and thiacloprid induced compound-specific transcriptional profiles, despite sharing a common mechanism of action. Furthermore, combined exposure resulted in gene expression patterns that differed from those observed with individual treatments. Together, these findings demonstrate that neonicotinoids can elicit divergent molecular responses, highlighting the importance of compound-specific toxicological assessment in non-target species.

## 1. Introduction

Neonicotinoid insecticides were introduced in the 1990s, when several organisms had already developed or were developing resistance to existing organophosphate, carbamate, and pyrethroid insecticides [1]. Currently, seven neonicotinoid compounds are available on the market: imidacloprid, nitenpyram, acetamiprid, thiamethoxam, thiacloprid, clothianidin, and dinotefuran [2]. The term neonicotinoids was proposed by Izuru Yamamoto to designate compounds structurally analogous to nicotine [3]. Nowadays, this class constitutes the most commercially available group of insecticides globally [4].

Neonicotinoids are among the fastest-growing classes of insecticides worldwide and are currently registered in more than 120 countries for use in approximately 140 crops [2,5]. Two of the main insecticides of this class are clothianidin (Sumitomo Chemical Takeda Agro Co./Bayer CropScience) and thiacloprid (Bayer CropScience). Both compounds were patented in the 1980s and introduced to the market in the 2000s. While clothianidin is classified as broad-spectrum insecticide, with more potent insecticidal activity when compared to other neonicotinoids, thiacloprid is considered less toxic to non-target species, with greater specific activity [6,7].

In general, the mechanism of action of neonicotinoids is based on their agonist activity at postsynaptic nicotinic acetylcholine receptors (nAChRs) [8,9], being more specific for insect nAChRs compared to mammalian and other vertebrate receptors [10,11,12,13]. This agonist activity promotes the neuronal depolarization and membrane hyperexcitation of insect neuronal membranes, resulting in paralysis and subsequent death [14].

Based on the properties of neonicotinoids and evidence obtained from animal studies, the World Health Organization (WHO) has classified them as moderately hazardous (class II) [6]. Even so, neonicotinoids continue to be used for insect control in a variety of settings, such as agriculture, commerce, households, and veterinary environments [12,14]. However, recently, the European Commission banned the use of neonicotinoid products due to their possible relationship with Colony Collapse Disorder (CCD), characterized by the sudden abandonment of hives by bees [8,9].

Added to this, several studies have already demonstrated that pesticides are harmful both to agricultural workers and to consumers through food and beverages [10,11], and they may be associated with various pathologies such as metabolic diseases [13,15] and immunotoxicity [16,17,18,19,20,21]. Other studies have also linked different pesticides to endocrine and reproductive disorders [22,23,24,25,26,27,28], neurotoxicity [29,30,31], hepatotoxicity [32], DNA damage [33], cancer [34,35], and gut microbiota dysfunction [36]. Consequently, these characteristics make it important to understand how these compounds can modulate the cell transcriptional profile.

## 2. Results

### 2.1. Multivariate Analysis Reveals Treatment-Driven Transcriptomic Signature

After filtering lowly expressed genes, 14,165 genes were retained for downstream analyses, corresponding to approximately 50% of the annotated transcriptome. To evaluate global transcriptional patterns across treatments, a principal component analysis (PCA) was performed using normalized expression values.

**Figure 1:**
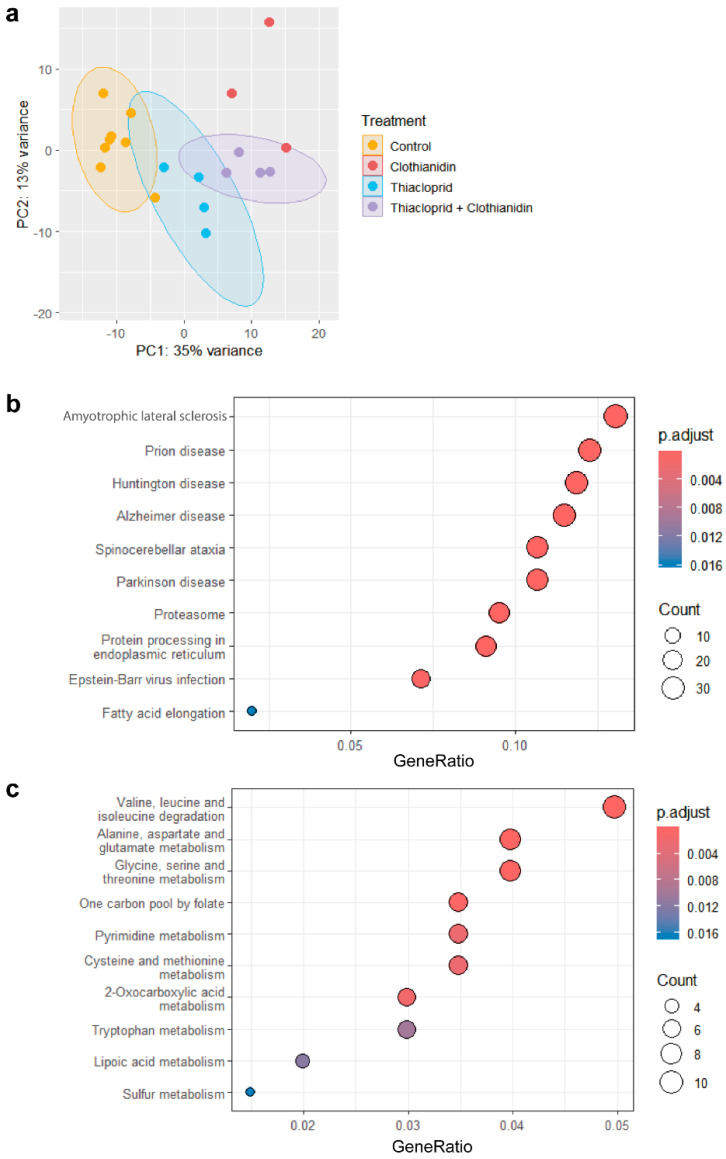
Multivariate transcriptomic analysis and functional enrichment associated with principal component 2 (PC2). (**a**) A principal component analysis (PCA) plot showing the distribution of control, thiacloprid, clothianidin, and mixture-treated samples based on variance-stabilized transcriptomic profiles. Samples clustering closer together exhibit more similar global gene expression patterns, whereas separation along principal components reflects transcriptomic divergence between treatment groups. PC2 was associated with treatment-related variation and was therefore selected for downstream correlation analyses. Group differences were confirmed by a PERMANOVA (R^2^ = 0.38; F = 3.07; *p* = 0.001), and an analysis of multivariate dispersion (betadisper) indicated no significant differences in within-group variability (*p* = 0.56). (**b**) A KEGG pathway enrichment analysis of the top 500 genes positively correlated with PC2. (**c**) A KEGG pathway enrichment analysis of the top 500 genes negatively correlated with PC2. Enrichment significance was determined using the Benjamini–Hochberg false discovery rate correction (adjusted *p* < 0.05).

The PCA revealed a clear separation among treatment groups (Figure 1a). PC1 explained 35% of the variation and primarily separated control samples from insecticide treatment groups, indicating a broad transcriptional response to neonicotinoid exposure. PC2 accounted for 13% of the variance and distinguished between individual treatments, suggesting that, although both neonicotinoids induced partially overlapping transcriptional responses, each compound also elicited distinct gene expression signatures.

To statistically validate group differences, a PERMANOVA was conducted, confirming that treatment significantly influenced the global transcriptomic profile (R^2^ = 0.38, F = 3.07, and *p* = 0.001). An analysis of multivariate dispersion (betadispersion) showed no significant differences among groups (*p* = 0.56), indicating that the observed separation was driven by differences in group centroids rather than heterogeneity within groups. Together, these findings indicate that neonicotinoid exposure promotes consistent and treatment-dependent transcriptomic remodeling across samples.

To investigate biological processes associated with PC2, genes showing the strongest positive and negative correlations with this component were subjected to KEGG enrichment analysis. The top ten genes positively correlated with PC2 were significantly enriched in pathways annotated in KEGG as neurodegeneration-related pathways, including amyotrophic lateral sclerosis, Alzheimer’s disease, and Parkinson’s disease (Figure 1b). Although these pathway annotations are disease-oriented, they comprise conserved molecular modules involved in mitochondrial function, oxidative metabolism, protein homeostasis and neuronal signaling. These findings suggest that PC2 captures transcriptional programs associated with neuronal and mitochondrial regulation under neonicotinoid exposure.

Conversely, the top ten genes negatively correlated with PC2 were enriched in metabolic pathways, including amino acid metabolism, one-carbon metabolism, and sulfur metabolism (Figure 1c). Collectively, these findings suggest that PC2 reflects a balance between neuronal-related and core metabolic transcriptional programs under neonicotinoid exposure.

### 2.2. KEGG Enrichment of Genes Modulated by Distinct Treatments

**Figure 2:**
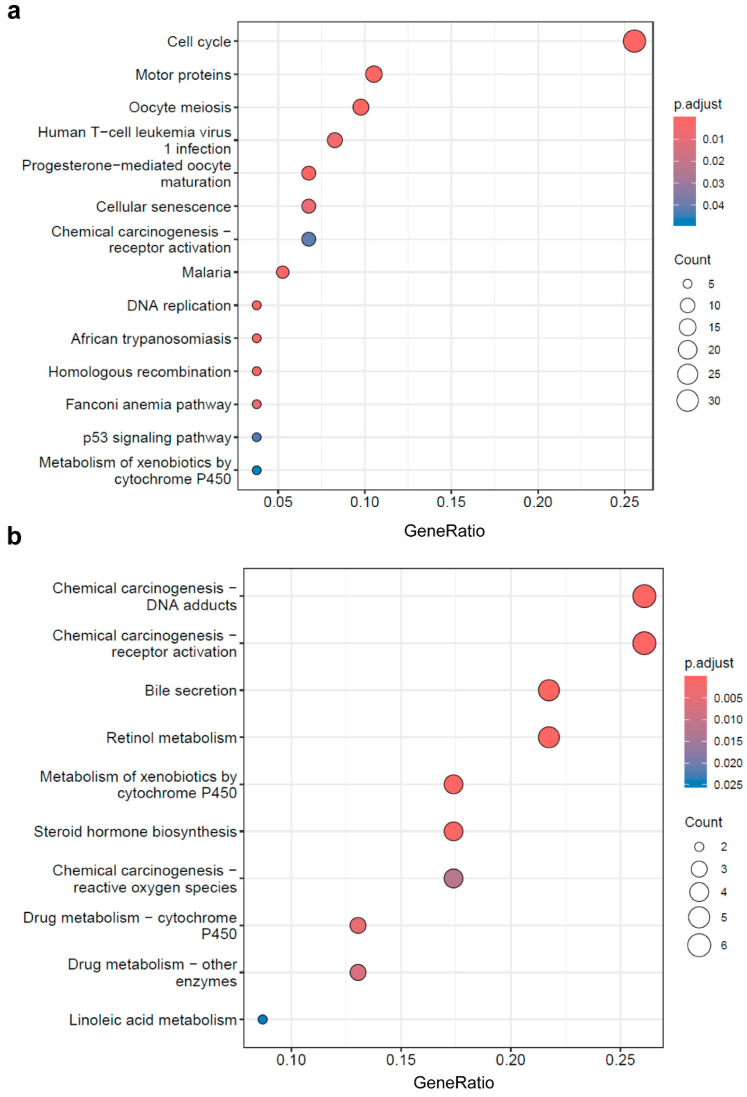
A KEGG pathway enrichment analysis of differentially expressed genes in response to neonicotinoid exposure. (**a**) Significantly enriched pathways identified in clothianidin-treated samples. (**b**) Significantly enriched pathways identified in thiacloprid-treated samples. The x-axis represents the GeneRatio, indicating the proportion of differentially expressed genes associated with each pathway. Dot size corresponds to the number of genes mapped to each pathway (count), and color indicates the adjusted *p*-value (Benjamini–Hochberg correction, adjusted *p* < 0.05).

To investigate functional signatures associated with each treatment, KEGG enrichment analysis was performed using differentially expressed genes (DEGs) identified for clothianidin, thiacloprid, and their combined treatment. For clothianidin-treated animals, enriched pathways included the cell cycle, cellular senescence, DNA replication, p53 signaling, chemical carcinogenesis receptor activation, and metabolism of xenobiotics induced by cytochrome P450 (CYP450) (Figure 2a). These annotations suggest the modulation of gene sets involved in cell cycle regulation and xenobiotic processing.

**Figure 3:**
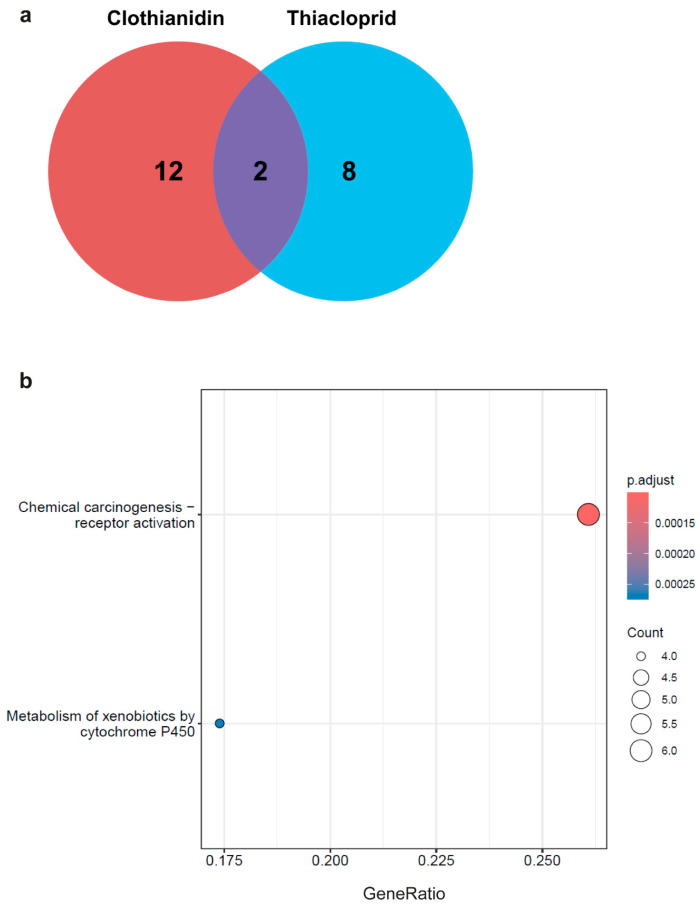
Shared KEGG pathway enrichment between neonicotinoid treatment. (**a**) A Venn diagram showing the number of significantly enriched KEGG pathways (adjusted *p* < 0.05) singularly or commonly identified in clothianidin- and thiacloprid-treated samples. (**b**) A dot plot representing KEGG pathways enriched in both treatments. The x-axis indicates the GeneRatio, reflecting the proportion of genes associated with each pathway relative to the total number of differentially expressed genes. Dot size corresponds to the number of genes mapped to each pathway (count), and color represents the adjusted *p*-value (Benjamini–Hochberg correction).

Thiacloprid treatment resulted in an enrichment in pathways related to chemical carcinogenesis (DNA adducts and receptor activation), drug metabolism, steroid hormone biosynthesis, and xenobiotic metabolism mediated by cytochrome P450 enzymes (Figure 2b), indicating the transcriptional modulation of genes involved in cell cycle regulation and detoxification-associated pathways. To evaluate shared functional responses between treatments, a Venn diagram was constructed to identify overlapping KEGG pathways (Figure 3a). Two pathways were commonly enriched across treatments: chemical carcinogenesis receptor activation and metabolism of xenobiotics mediated by CYP450 (Figure 3b). This overlap indicates that both neonicotinoids modulate conserved gene sets associated with xenobiotic sensing and metabolic processing.

### 2.3. Treatments Induce Distinct Patterns of Gene Expression

**Figure 4:**
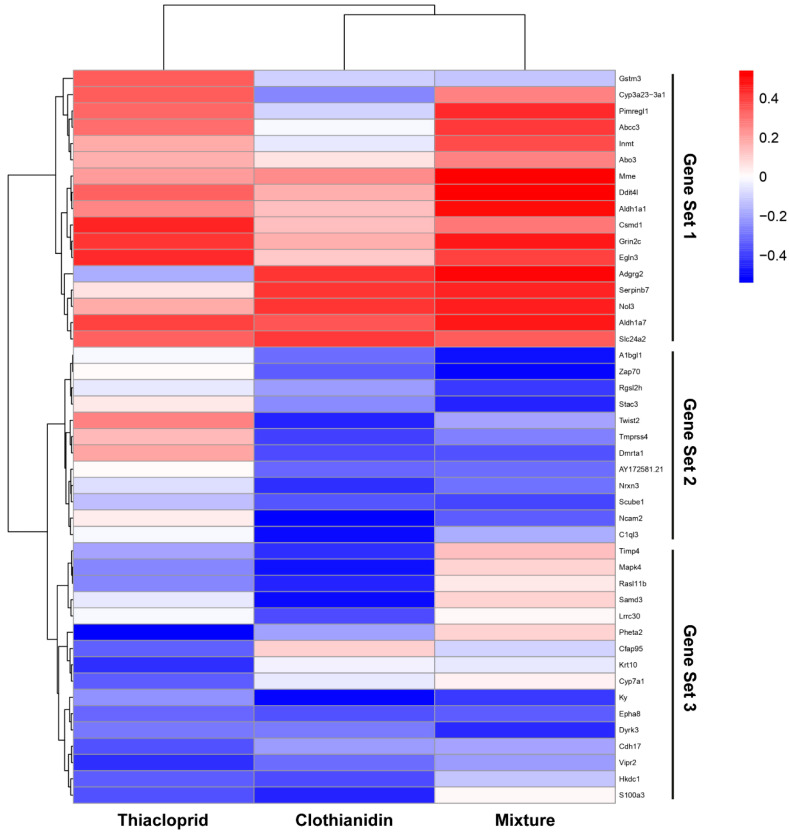
A correlation heatmap between gene expressions patterns and treatments using Spearman’s correlation coefficient. Warmer colors indicate positive correlations between gene expression levels and treatment conditions, whereas cooler colors indicate negative correlations. Genes with similar correlation profiles across treatments were grouped by hierarchical clustering using Ward’s method, allowing for the identification of coregulated expression patterns and treatment-associated transcriptional signatures.

To further characterize treatment-associated transcriptional patterns, a correlation heatmap was generated using Spearman’s correlation coefficients based on the thirty most strongly modulated genes from each treatment group (Figure 4). Hierarchical clustering revealed distinct groups of genes exhibiting coordinated expression responses across neonicotinoid exposures.

Gene set 1 was predominantly composed of genes associated with detoxification and cell proliferation and displayed strong positive correlations with all treatments, indicating coordinated transcriptional activation. Thiacloprid induced broad upregulation across this cluster, whereas clothianidin showed stronger associations with proliferation-related genes. The mixture treatment produced a similar activation profile, suggesting the partial convergence of transcriptional response between individual and combined exposures.

Gene set 2 consisted mainly of genes related to immune response, cell proliferation, and cell adhesion and generally exhibited weak or negative correlations with treatments, suggesting transcriptional repression. Clothianidin and the mixture treatment showed the strongest negative correlation within this cluster, whereas thiacloprid induced comparatively weaker effects. These findings indicate that distinct neonicotinoids may differentially modulate pathways associated with immune and cellular regulatory processes.

Finally, Gene set 3 included genes associated with cytoskeleton organization and cell proliferation and showed an overall downregulation following exposure to individual insecticides. In contrast, the mixture treatment produced weaker or partially reversed correlation patterns, suggesting that combined exposure induces transcriptional responses distinct from those elicited by either compound alone. Although formal interaction models were not applied in this study, these findings support the possibility that co-exposure modifies the regulation of specific gene networks compared to single-compound treatments.

## 3. Discussion

The present study aimed to investigate whether neonicotinoid insecticides sharing the same mechanism of action exhibit similar toxicological effects based on toxicotranscriptomic analyses. Clothianidin and thiacloprid were selected as model neonicotinoids for this study. Interestingly, despite sharing the same mechanism of action, namely agonism in nAChRs, distinct responses were observed rather than a common toxicological pathway.

From an agricultural perspective, understanding pesticide stress biology extends beyond target pest control, encompassing broader ecological consequences for non-target species and crop-associated organisms. Pesticide exposure can trigger conserved molecular stress pathways, including xenobiotic metabolism, oxidative stress signaling, and transcriptional reprogramming, responses that have been characterized across distinct insecticide classes with different modes of action [37]. In parallel, insecticide exposure has been shown to modulate post-transcriptional regulatory networks, including miRNA-mediated gene regulation, which may affect development, reproduction, and metabolic homeostasis in non-target organisms [38]. These molecular frameworks are relevant to crop-associated toxicological responses, as they highlight how chemically distinct compounds can converge on shared stress biology while simultaneously eliciting compound-specific transcriptional signatures, as observed in the present study.

Although designed as a selective insecticide, clothianidin has been associated with cytotoxic effects in non-target species [11], including disturbances in cell cycle regulation and the activation of stress response pathways. Experimental evidence indicates that exposure to neonicotinoids can induce cell cycle arrest, commonly at the G1/S or G2/M checkpoints, potentially mediated by DNA damage [39] and the subsequent activation of p53 signaling [40]. The p53 pathway plays a central role in regulating genes involved in cell cycle progression [41] and cytoskeletal dynamics, including motor proteins such as kinesins and dyneins, whose dysfunction may impair mitotic processes [42,43,44,45]. Moreover, sustained p53 activation is closely linked to the induction of cellular senescence [46], characterized by irreversible growth arrest and a pro-inflammatory secretory phenotype.

Studies on neonicotinoids, including clothianidin, demonstrate increased oxidative stress and mitochondrial dysfunction, characterized by elevated reactive oxygen species (ROS) production, the loss of mitochondrial membrane potential, and the activation of apoptotic signaling pathways [18,47,48,49], which are well-known upstream triggers of p53 activation and cellular senescence. These findings suggest a mechanistic axis involving cellular stress, the disruption of cell cycle control, the impairment of motor proteins, and the activation of p53 signaling, although further studies are required to clarify these interactions in human-relevant systems.

Interestingly, neonicotinoid insecticides undergo relatively conserved biotransformation in mammals, primarily involving phase I oxidative reactions mediated by cytochrome P450 enzymes, followed by phase II conjugation pathways [26]. A key metabolic feature is the biotransformation of thiamethoxam into clothianidin, a pathway consistently demonstrated in both experimental [50,51] and environmental studies [52,53]. Clothianidin itself may undergo further metabolism, including denitration and conjugation reactions [54]. However, its relative persistence [55] contributes to its frequent detection in environmental matrices [56] and human biomonitoring samples [57]. Therefore, the formation of clothianidin represents a toxicologically relevant metabolic route, as it may prolong systemic exposure to biologically active neonicotinoid-derived compounds.

In addition, thiacloprid has been increasingly associated with hepatic metabolic disturbances that may impact pathways such as bile secretion, retinol metabolism, and steroid hormone biosynthesis. Experimental and omics-based studies indicate that thiacloprid exposure disrupts hepatic metabolic pathways, including bile acid and cholesterol metabolism, as demonstrated by multiomics analyses showing altered bile acid homeostasis and liver injury [58,59]. These processes are closely linked to hepatobiliary transport systems, including ATP-binding cassette (ABC) transporters, which play a central role in bile secretion and xenobiotic excretion [60]. Disruption of these systems may impair bile flow and promote the hepatic accumulation of toxic intermediates. In parallel, the modulation of retinol metabolism has been reported following neonicotinoid exposure [61,62,63], potentially interfering in enzymes such as retinol dehydrogenases, retinal oxidases [62] and cytochrome P450 isoforms responsible for retinoic acid synthesis and degradation [64,65]. Such alterations may affect vitamin A homeostasis and downstream signaling pathways.

Furthermore, neonicotinoids, including thiacloprid, have been shown to exert endocrine-disrupting effects through multiple mechanisms [66,67,68], potentially affecting steroid hormone biosynthesis through interference with steroidogenic enzymes and nuclear receptor signaling pathways [69,70]. Although further studies are required to elucidate these interactions in humans, these data support a mechanistic framework in which thiacloprid exposure may disrupt liver function and systemic homeostasis through coordinated effects on bile secretion, retinoid metabolism, and steroidogenesis.

Clothianidin and thiacloprid modulated gene sets enriched in KEGG pathways annotated as chemical carcinogenesis-related processes. These pathways include molecular modules associated with oxidative stress responses, xenobiotic metabolism, DNA damage signaling, and cellular stress regulation. These early molecular events are frequently represented in carcinogenesis-related Adverse Outcome Pathway (AOP) frameworks. Experimental studies indicate that neonicotinoids can induce oxidative stress and mitochondrial dysfunction, resulting in the excessive production of ROS [18,48,71] and oxidative DNA damage, including double-strand breaks marked by γH2AX [72,73,74]. These early key events are central to carcinogenesis AOPs, as they trigger the activation of the p53 pathway, which regulates cell cycle arrest, DNA repair, and apoptosis in response to genotoxic stress [75,76]. Persistent or improperly repaired DNA damage may lead to genomic instability, a hallmark of cancer, particularly when error-prone repair mechanisms are engaged [77,78,79].

In parallel, the modulation of xenobiotic-metabolizing enzymes, such as cytochrome P450s, can influence the metabolic activation of procarcinogens, representing a molecular initiating event in several chemical carcinogenesis AOPs [80,81]. These enzymes convert inert compounds into reactive intermediates capable of inducing DNA damage and initiating cell transformation [82]. Within this framework, sustained oxidative stress and dysregulated stress response signaling may shift cellular outcomes from protective apoptosis toward survival with accumulated mutations, potentially contributing to molecular environments associated with cellular transformation [83,84].

Even though direct epidemiological evidence linking neonicotinoids to cancer incidence in humans remains limited, the convergence of biomonitoring, endocrine disruption, and genotoxicity data supports a biologically plausible framework in which chronic exposure to clothianidin and thiacloprid may contribute to molecular processes associated with cellular stress and toxicological responses. Epidemiological and biomonitoring studies indicate that human exposure to neonicotinoid insecticides is widespread [85,86] and may be associated with endocrine disruption [87] and pathways relevant to chemical carcinogenesis [88]. Large-scale exposure assessments have detected neonicotinoid metabolites in human urine across different populations, supporting continuous low-level exposure [89,90].

## 4. Materials and Methods

### 4.1. Data Source and Experimental Design

Publicly available RNA sequencing data were retrieved from the Gene Expression Omnibus (GEO) database under accession number GSE153986 [58]. The original study evaluated liver transcriptomic responses in five-week-old female Wistar rats treated with neonicotinoid insecticides thiacloprid and clothianidin and their combination. Animals were treated by oral gavage for 28 days using a repeated-dose administration protocol. Test compounds were suspended in 0.5% aqueous carboxymethyl-cellulose and administered at a dosing volume of 7.5 mL/kg body weight. Control animals received vehicle only (0.5% of aqueous carboxymethyl-cellulose in deionized water). Experimental groups included the control (n = 8), thiacloprid-treated (n = 4), clothianidin-treated (n = 4), and combined treatment groups (n = 4).

Total RNA sequencing was performed in the original study using the TruSeq Stranded total RNA Sample Preparation Kit (Illumina, San Diego, CA, USA) with 100 ng RNA per sample. Sequencing was conducted on the Illumina NovaSeq 6000 platform, generating approximately 50–160 million paired-end 100 bp reads per sample. Raw sequencing quality was assessed using FastQC version 0.11.5, and adapter trimming was performed using Skewer version 0.2.2. Reads were aligned to the rat reference genome (Rnor_5.0) using STAR version 2.5.2b [58].

Raw sequencing data were downloaded and processed in R Studio (version 4.3.1), and downstream analyses were conducted using BioConductor packages. Batch effect assessment was performed through exploratory multivariate analyses, including PCA and dispersion analyses. No evident clustering patterns suggestive of a technical batch effect were observed among samples.

### 4.2. Differential Gene Expression Analysis

Differential gene expression analysis was conducted using the DESeq2 package (version 1.42.1), which models count data based on the negative binomial distribution and applies shrinkage estimation for dispersion and fold change parameters [91]. Normalization was conducted using the median of ratios method implemented in DESeq2 to account for sequencing depth and RNA composition biases.

For each comparison, a generalized linear model was fitted to estimate log2 fold changes between the control and treatment groups. Pairwise comparisons were performed between: thiacloprid vs. control, clothianidin vs. control, and combined treatment (mix of thiacloprid and clothianidin) vs. control. Statistical significance was evaluated using the Wald test. The resulting *p*-values were adjusted for multiple tests using the Benjamin–Hochberg (BH) false discovery rate (FDR) correction. Genes were considered significantly differentially expressed when they met the following criteria: adjusted *p*-value (padj) < 0.05 and absolute log2 fold change (|log2FC|) ≥ 1. These thresholds were selected based on commonly adopted criteria in transcriptomic studies to balance statistical significance and biological relevance. Both upregulated and downregulated genes were retained for downstream functional enrichment analyses.

### 4.3. Gene Filtering

To ensure the inclusion of reliably expressed genes in downstream analyses, a filtering step was applied prior to PCA. Genes were retained if they presented at least 10 raw counts in a minimum of three samples. Genes not meeting this criterion were excluded from further analyses.

### 4.4. Principal Component Analysis (PCA)

Variance stabilizing transformation (VST) counts were obtained using the DESeq2 package to reduce heteroscedasticity. Principal component analysis was performed to estimate global transcriptomic variations among treatment groups. The contribution of principal component 2 (PC2) was further investigated to assess treatment-related changes in transcriptional signatures.

To statistically evaluate differences in global gene expression profiles among groups, a permutational multivariate analysis of variance (PERMANOVA) was conducted using Bray–Curtis dissimilarity matrices with 999 permutations. The homogeneity of the dispersion was assessed prior to the PERMANOVA to ensure that significant differences were not driven by unequal group dispersion.

For gene ranking associated with principal component 2 (PC2), loadings were extracted from the PCA performed using the plotPCA function in DESeq2 based on the VST count. Genes were ranked according to their loading magnitude, and the top 500 positively and negatively associated genes were selected for subsequent enrichment analyses.

In the final step, genes significantly correlated with PC2 were identified using correlation analysis. Significant correlations were defined as r ≥ 0.4 and padj < 0.05, controlling for multiple testing using the BH procedure. The correlation coefficient was estimated for each gene across the samples (control and treatment groups).

### 4.5. Functional Enrichment Analysis with KEGG Pathway

Functional enrichment analysis was performed using the clusterProfiler package (version 4.10.1) [92]. Differentially expressed genes were converted from ENSEMBL identifiers to ENTREZ IDs using the org.Rn.eg.db (version 3.18.0) annotation database [93] with genome-wide annotation for rats.

Kyoto Encyclopedia of Genes and Genomes (KEGG) pathway [94] enrichment analysis was executed using the enrichKEGG function. Pathways with padj < 0.05 were considered significantly enriched. Separate enrichment analyses were conducted for: upregulated genes, downregulated genes, PC2-positive genes, and PC2-negative genes. Finally, shared enriched pathways between thiacloprid and clothianidin treatments were identified using set intersection analyses.

### 4.6. Visualization and Statistical Analysis of Differential Gene Expression

Dot plots of enriched KEGG pathways were generated with the clusterProfiler method and visualization function [92]. Gene selection for the heatmap was based on the log2 fold change (log2FC) and adjusted *p*-values calculated using the Benjamini–Hochberg method. The top 30 most strongly modulated genes (up- or downregulated) for each treatment were selected for visualization. In the second step, genes lacking valid SYMBOL or ENSEMBL annotation were removed. Genes overlapping between treatments were merged to simplify visualization. Finally, the heatmap was generated using the pheatmap package (version 1.0.13) with hierarchical clusterization (Ward’s method). All statistical analyses were performed in R Studio, and graphical representations were generated using the ggplot2 package (version 4.0.1).

## 5. Conclusions

In summary, it was demonstrated that despite sharing the same mechanism of action, clothianidin and thiacloprid induce distinct patterns of gene expression modulation. While clothianidin predominantly modulated gene sets associated with cell cycle regulation, motor proteins and cellular senescence, thiacloprid predominantly affected pathways related to bile secretion and metabolic processes. Both pesticides commonly modulated gene sets enriched in pathways related to xenobiotic metabolism, cellular stress responses, and chemical carcinogenesis-associated molecular processes.

Importantly, these findings are supported by a robust and integrative analytical framework based on publicly available RNA-seq data, including multivariate analysis (PCA and PERMANOVA), differential gene expression with stringent thresholds (adjusted *p*-value and |log2FC|), functional enrichment, and correlation-based approaches. Therefore, these results reinforce the importance of evaluating pesticides in non-target species, highlighting that compounds sharing similar mechanisms of action may still induce distinct responses depending on exposure conditions.

The present study was based on the reanalysis of publicly available transcriptomic data, and no independent validation assays, such as RT-qPCR, protein-level analyses, or the use of oxidative stress biomarkers, were performed. Therefore, the observed transcriptomic signatures and pathway enrichment results should be interpreted as molecular associations and functional trends rather than direct evidence of pathological outcomes. In addition, formal interaction analyses or combined toxicity models were not employed for the mixed exposure group. Future studies integrating transcriptomics with functional and biochemical validation approaches are necessary to further clarify the biological relevance of these molecular alterations.

## Data Availability

The datasets analyzed throughout this study are publicly available in the Gene Expression Omnibus (GEO) repository under accession number GSE153986 [57]. All the bioinformatic scripts used for data processing and analysis for this work are available from the corresponding author upon reasonable request.
